# Developments in ^177^Lu-based radiopharmaceutical therapy and dosimetry

**DOI:** 10.3389/fchem.2023.1218670

**Published:** 2023-07-31

**Authors:** Siju C. George, E. James Jebaseelan Samuel

**Affiliations:** ^1^ Radiation Oncology Department, Miami Cancer Institute, Baptist Health, Miami, FL, United States; ^2^ Department of Physics, School of Advanced Sciences, Vellore Institute of Technology, Vellore, India

**Keywords:** ^177^Lu, absorbed dose, patient-specific dosimetry, dose calculations, imaging, calculation methods, clinical trials, contemporary developments

## Abstract

^177^Lu is a radioisotope that has become increasingly popular as a therapeutic agent for treating various conditions, including neuroendocrine tumors and metastatic prostate cancer. ^177^Lu-tagged radioligands are molecules precisely designed to target and bind to specific receptors or proteins characteristic of targeted cancer. This review paper will present an overview of the available ^177^Lu-labelled radioligands currently used to treat patients. Based on recurring, active, and completed clinical trials and other available literature, we evaluate current status, interests, and developments in assessing patient-specific dosimetry, which will define the future of this particular treatment modality. In addition, we will discuss the challenges and opportunities of the existing dosimetry standards to measure and calculate the radiation dose delivered to patients, which is essential for ensuring treatments’ safety and efficacy. Finally, this article intends to provide an overview of the current state of ^177^Lu- tagged radioligand therapy and highlight the areas where further research can improve patient treatment outcomes.

## 1 Introduction

Radioligand therapy (RLT) delivers radiation to target cells anywhere in the body by harnessing the radioactive atoms’ power. Radioligands emerged as a promising treatment method by targeting cancer cells while sparing healthy tissue. A high radiation dose can be delivered directly to cancer cells using radiolabeled peptides that selectively seek the receptors present in the tumor. One of the significant benefits of treatments with ^177^Lu-based radioligands is that they can be applied to both primary tumors and metastatic cancers, Sgouros et al. (2020). Another advantage of ^177^Lu-based treatments is that they tend to be relatively low in toxicity. In contrast to traditional chemotherapy, ^177^Lu-based treatment minimally damages healthy tissues, which conventional chemotherapy may otherwise damage. Therefore, patients experience fewer side effects and a better quality of life from radioligand-based treatments, Sartor et al. (2021). Access to such therapy is essential for patients suffering from advanced-stage cancer, where options for conventional treatments may be limited. It has been found that ^177^Lu-based treatments yield significant positive results in treating gastroenteropancreatic neuroendocrine tumors (GEP-NET), including pancreatic neuroendocrine tumors that express somatostatin receptors (SSTR) and patients with prostate-specific membrane antigen (PSMA) positive metastatic castration-resistant prostate cancer (mCRPC).

### 1.1 Chemistry of Lu-177

**FIGURE 1:**
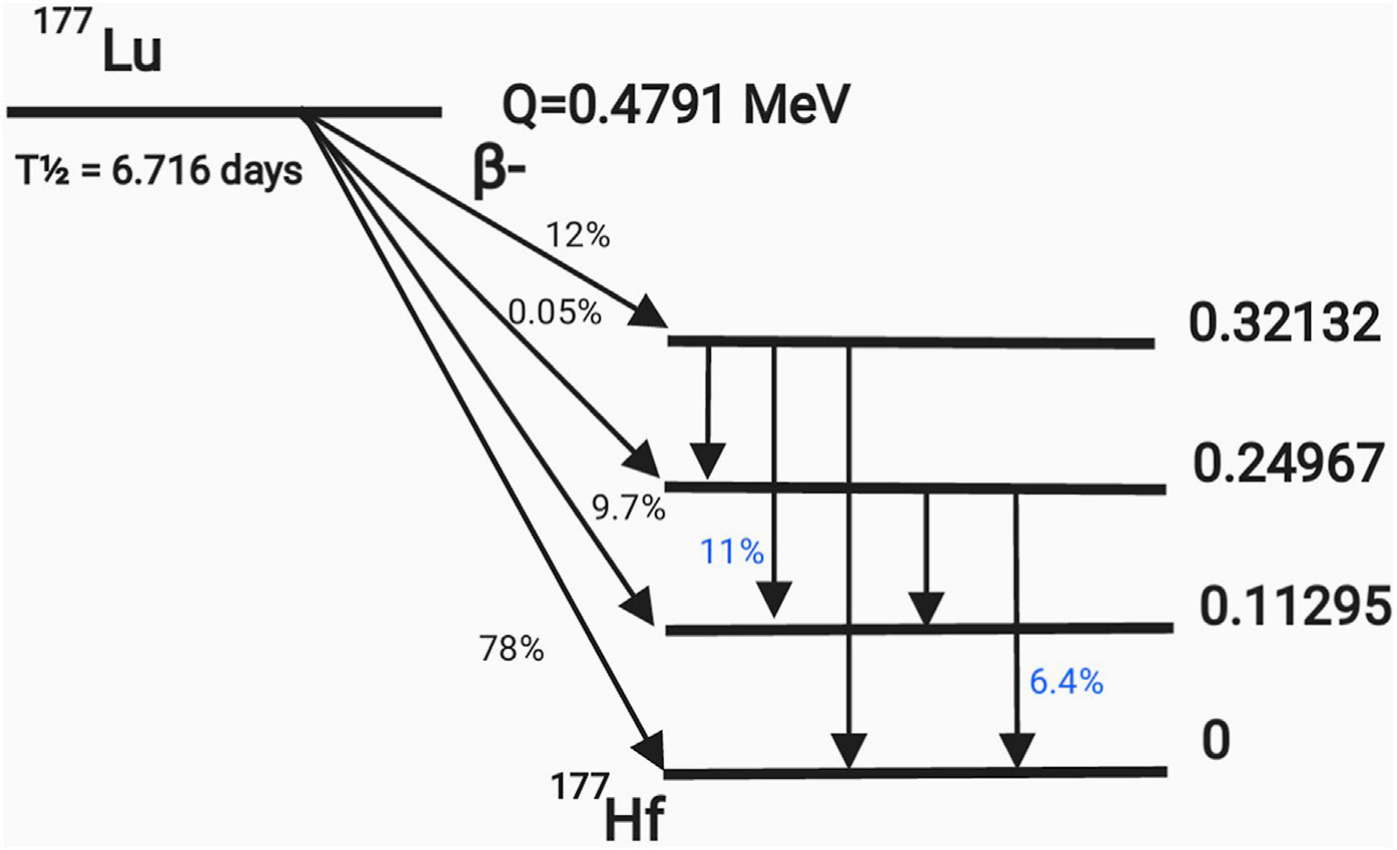
Decay scheme of ^177^Lu. The probabilities of gamma emission are shown in blue, while the probabilities of beta emission are in black.

In recent years ^177^Lu has become a popular radionuclide due to its convenient half-life of 6.647 days, its +3 oxidation state for easy radio-labeling, and the energy of the emitted photons and electrons. The half-life is long enough that transportation, storage, and delivery are not a challenge but short enough that non-targeted organs are safe from high doses, Sergey Gudkov (2015). ^177^Lu is both a *β* and *γ* emitter. This dual emission allows ^177^Lu as an appropriate agent for treatment and imaging. The main beta energies emitted are 497 keV (78%), 384 keV (9.7%), and 176 keV (12%). The main gamma photons emitted are 113 keV (6.4%) and 208 keV (11%). Figure 1 displays the decay scheme of ^177^Lu. Beta particle energies allow ideal soft tissue penetration of 670 μm, sufficient to kill the tumor cell, but leave surrounding normal tissue with limited effects associated with radiation exposure.

Although Lutetium is classified as a Lanthanide series element, it does not show all the chemical properties associated with this series. However, the predominant ionic character (ionic potential) of the Lu^3+^ ion enables the formation of robust aggregates with stable ligands in an aqueous solution, which is most commonly used to prepare radiopharmaceuticals for injection, Parus et al. (2015). While ^177^Lu may have better nuclear properties than other therapeutic radionuclides, the clinical application of this radioisotope is currently limited to only two Food and Drug Administration (FDA) approved radiopharmaceuticals. Several research groups are investigating chelators, ligands, and complexes that can deliver radiopharmaceuticals safely and effectively to various target areas in the human body.

### 1.2 Click chemistry applied in medicine

“Click chemistry” is a term used to describe a set of highly selective and efficient chemical reactions that attach functional groups to a targeting molecule. This innovative approach has been instrumental in advancing scientific research. In recognition of their contributions to click chemistry, Barry Sharpless, Morten Meldal, and Carolyn Bertozzi were awarded the 2022 Nobel Prize in Chemistry, Nobel (2022).

Click chemistry has several applications, including ^177^Lu chelation. This involves attaching ^177^Lu to a targeting molecule through a chemical reaction. Compared to other methods, ^177^Lu chelation offers advantages such as rapidity, efficiency, and specificity in connecting ^177^Lu to various targeting molecules. These click reactions are highly selective, attaching ^177^Lu to a specific site in the targeting molecule and minimizing the risk of nonspecific binding, Parus et al. (2015).

Click chemistry enables the radiolabeling of the chelator separately and subsequent connection to the binding molecule instead of radiolabeling the complex of the chelator-targeting molecule. This approach offers an advantage, as the radiolabeling conditions could damage the targeting molecule. To illustrate, the process of attaching ^177^Lu-DOTA-N3 to the dibenzocyclooctyne (DBCO)-bearing CTT1298 PSMA inhibitor cores was accomplished by applying click chemistry. This process facilitated the creation of CTT1401 and CTT1403, Choy et al. (2017).

## 2 Molecular radiation therapy (MRT)

The MRT method employs radiolabeled molecules that specifically bind to molecules or other targetable biological materials, including peptides, antibodies, and small molecules that are overexpressed in the microenvironment of cancer cells, Sergey Gudkov (2015). By delivering radioligands directly to cancer cells, taking advantage of the chemical affinity of the overexpressed receptor molecules in the cancer microenvironment, a known dose is given to the tumor. Typically, radiopharmaceuticals bind to specific receptors or proteins on the surface of tumors targeted for treatment. After the molecule binds to the radiolabeled molecule, the cancer cell engulfs it, allowing selective radiation delivery that kills the tumor cell while limiting damage to normal cells. Radionuclide therapy using radiolabeled peptides has become one of the most common methods for treating tumors, consisting of various conglomerations that arise from cells that release hormones in response to signals from the nervous system, Sjogreen Gleisner et al. (2022).

### 2.1 Brief history of radio-pharmaceuticals

Some historically significant developments and their clinical implications are briefly discussed below. These developments contributed to other radiopharmaceuticals’ evolution and wide use in medical practice. Pandit-Taskar et al. (2021), presented a systematic analysis of clinically available radiopharmaceuticals, indications for use, administration methods, dosages, and dosimetry requirements. Eleven radiopharmaceutical therapies (RPT) are available for clinical use for various indications. Only four of the 11 FDA-approved drugs require dosimetry, while others use empirical methods to determine individual patients’ drug doses.

Radiopharmaceuticals have been used to treat hyperactive thyroid disease since the early 1940s when ablative doses of ^131^I were administered to reduce thyroid activity. The thyroid gland naturally accumulates iodine from the body. To successfully treat thyroid cancer, as much thyroid tissue as possible must be removed during surgery. After adequate postoperative recovery, the patient receives a prescribed amount of radioactive iodine, which accumulates preferentially in the remaining thyroid cells, destroying them. Hematology-related RPT trials began following successful thyroid treatments. Clinical trials with ^131^I tositumomab were initiated in 1990 to treat relapsed or refractory and transformed low-grade Non-Hodgkins Lymphomas (NHL). Due to NHL’s inherent radiosensitivity and anti-CD20 antibody therapy’s known efficacy, radioimmunotherapy regimens are ideal for low-grade NHL, Fisher et al. (2005). The US FDA approved Zevalin^®^ (^90^Y ibritumomab tiuxetan), the first conjugated antibody for treating NHL, on 19 February 2002 (approval is still pending for the EEC).

Selective Internal Radiation Therapy (SIRT) destroys liver tumors. This modality differs from RLT, but imaging and dosimetry characteristics are relevant for understanding the therapeutic outcome. While SIR-Sphere^®^ comprises resin microspheres coated with ^90^Y, TheraSphere^®^ comprises insoluble glass microspheres covered with ^90^Y. SIR-Spheres were approved by the FDA in 2002 for treating non-resectable liver tumors, and ^90^Y TheraSphere was approved later for treating hepatocellular carcinoma. In both cases, spheres are injected directly into the tumor-bearing liver lobes for radio ablation, Lawhn-Heath et al. (2022).

Bone metastasis is one of the most common routes cancer spreads from a primary site. As cancer cells invade bone tissue, they destroy the bone, which the body replaces through a process called bone turnover. An injectable radioactive element that mimics calcium may be able to access the impacted bone through the bone turnover process and attack cancer cells within bone metastases. Even though ^89^Sr-chloride and ^153^Sm-ethylenediamine tetra provided significant pain relief, little is known about their effect on patient survival. RPT was revolutionized by the introduction of *α* emitting ^223^Ra Cl_2_, which expanded its use from palliation alone to treating bone metastases, Pandit-Taskar et al. (2021). In May 2013, the FDA approved the injection of Xofigo^®^ (Bayer Pharmaceuticals) to treat CRPC patients with symptomatic bone metastases without visible visceral metastatic disease.

### 2.2 Dosimetric limitations and the traditional “one size fits all” approach used in RPT

Several factors lead to radiopharmaceuticals being administered as a one-size-fits-all treatment. Compared to patient-specific calculations, this process is more straightforward and practical, as it does not require individualized dosimetry calculations and is less time-consuming. The dose administered to specific organs or tissues may be more critical than the total dose administered to patients. Therefore, it is essential to administer small enough doses with a fixed quantity to ensure that the dose limits of individual organs are not exceeded. This is because radiopharmaceuticals often accumulate based on the physiological properties of a particular organ or tissue. Implementing patient-specific dosimetry can be challenging due to regulatory or institutional constraints, including limiting the amount of radioactivity an individual can receive and manage, Danieli et al. (2022).

However, a one-size-fits-all approach has numerous disadvantages. Patient undertreatment or overtreatment is a significant concern. The absorption and elimination of radiopharmaceuticals may differ widely among individuals due to anatomy, physiology, and metabolic differences. When a fixed dose is used, the target tissue may not receive an optimal radiation dose, resulting in a suboptimal therapeutic effect or unwanted toxicity. Furthermore, fixed quantities do not consider the underlying health status of the patient, which may affect their tolerance to radiation. Individualized dosimetry can address these concerns, optimizing treatment outcomes and minimizing side effects, Pandit-Taskar et al. (2021).

Due to the limited availability of quantitative imaging techniques and accurate dosimetry models, dosimetry was not a significant part of radiopharmaceutical therapy in the 90s. Radiation exposure to various organs was not evaluated as accurately as today due to limitations in imaging technology and computational methods. Due to this, many radiopharmaceuticals were developed and used based on their observed clinical outcomes without precise dosimetric calculations. Dosimetry has become increasingly crucial to radiopharmaceutical development and clinical practice with the advent of sophisticated imaging techniques and computing tools, Danieli et al. (2022).

### 2.3 Role of patient-specific dosimetry in FDA-approved treatments and upcoming trials

Patient-specific dosimetry is an essential aspect of RPT, as it accurately determines the radiation dose that a patient will receive from a specific treatment. This is important because the therapeutic benefit of radiopharmaceuticals is directly related to the amount of radiation delivered to the target area, and accurate dose measurement allows correlation with treatment outcomes. Additionally, dosimetry helps to ensure that the radiation dose is within the safe organ tolerance dose limits for the patient, minimizing risk of adverse effects. The origin of current organ absorbed dose limits is mainly based on external beam radiation (EBRT) data and extensive quantitative analysis of typical tissue effects in the clinic. This analysis was published in the International Journal of Radiation Oncology—Biology—Physics (IJROBP) (March 2010, Volume 76, Issue 3, Supplements S1-S160), based on a review and meta-analysis of the literature from many decades of experience with external beam radiation, Bentzen et al. (2010).

The European Association of Nuclear Medicine (EANM) dosimetry committee provided the data and methods available for ^177^Lu-based dosimetry. It comprehensively compiles an overview of dosimetry data for ^177^Lu-labeled therapies and summarizes current knowledge of radiation-induced side effects and dose-effect relationships, Sjogreen Gleisner et al. (2022).

The EANM position paper provided a guide on interpreting the statements of the directive for nuclear medicine treatments outlined in Article 56 of the Council directive 2013/59/EURATOM (basic safety standards for nuclear medicine therapy), Konijnenberg et al. (2021). To comply with the optimization principle of the directive, EANM proposed three separate recommendations based on the prescribing, recording, and reporting of absorbed doses in radiotherapy defined by the International Commission on Radiation Units and Measurements (ICRU).

1. As a general rule, standardized treatments require that the dose administered is within 10% of the intended activity.
2. Non-standardized treatments, including patient-specific dosimetry, require recording and reporting the absorbed dose to OARs and, optionally, to treatment regions.
3. An administered activity is calculated to deliver a desired absorbed dose to a treatment region or OAR in a research setting. New dosimetry methodologies should be developed to predict response or toxicity better.
  Nuclear medicine treatments and patient-specific dosimetry are highly standardized and regulated in Europe, Lassmann et al. (2021). The situation is different in the United States of America and other countries where ^177^Lu-based RLTs are becoming more prevalent in mainstream cancer treatments.

## 3 The evolution of ^177^Lu-based radiopharmaceutical therapy

**FIGURE 2:**
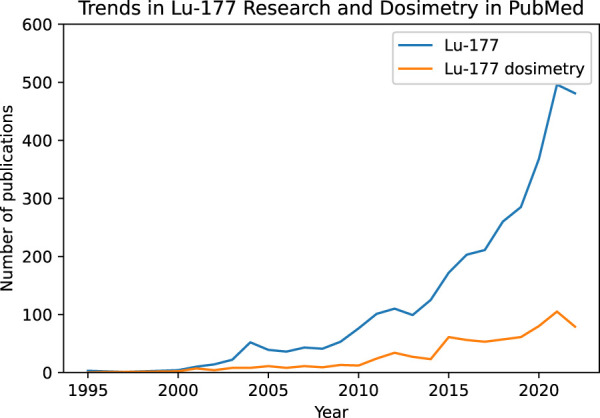
Research interest in ^177^Lu over the years in PubMed since 1995 until 2022. A peak of 496 publications about ^177^Lu occurred in 2021 (blue). Adding “Dosimetry” to the search reduced the total number of papers published with a maximum of 105 papers in 2021 (orange).

The development of ^177^Lu-based cancer treatments has been extensively investigated over the last two or three decades. According to a PubMed search, 3,312 articles were published on ^177^Lu, while 755 articles were published on ^177^Lu dosimetry from 1995 to 2022. Figure 2 shows the number of articles published per year, demonstrating the growth of scientific interest in ^177^Lu and its dosimetry, Aguirre and George (2023).

### 3.1 Currently available treatments in clinical settings

The NETTER-1 study randomized patients to receive ^177^Lu-Dotatate at a dose of 7.4 GBq every 8 weeks (four intravenous infusions). It was demonstrated in 2017 that ^177^Lu-Dotatate in combination with long-acting octreotide (20–30 mg) significantly improved progression-free survival in patients with these tumors compared to high-dose long-acting octreotide alone (60 mg), Strosberg et al. (2017). In 2018, the FDA approved targeted radiation treatments of NET with the radiopharmaceutical LUTATHERA^®^ (^177^Lu-DOTATATE), a medicine used to treat adults with GEP-NETs in the foregut, midgut, or hindgut that are positive for the hormone receptor somatostatin, AAA (2023).

Phase 3 trial of ^177^Lu-DOTATE for neuroendocrine tumors of the midgut compared two groups, one treated with ^177^Lu-Dotatate plus repeatable long-acting octreotide (LAR) and the other with LAR octreotide alone. The results showed a significant difference in the progression-free survival rate (PFS) of 65.2% vs. 10.8% after 18 months of therapy. The response rate was 18% vs. 3%, respectively, Strosberg et al. (2017). While this therapy has shown significant efficacy in treating NET, it can cause dose-limiting toxicities in organs at risk (OAR), such as the kidney and bone marrow, Wahl et al. (2021).

PSMA is a type II transmembrane protein expressed predominantly in prostate cancer cells, Rajasekaran et al. (2005). Multiple clinical trials have used a variety of PSMA peptides in men with mCRPC. Among these, the most promising is ^177^Lu PSMA-617, which became FDA-approved in May 2022 and was branded under the name PLUVICTO^®^ used in patients treated with androgen-receptor pathway inhibition and taxane-based chemotherapy. In the VISION trial, ^177^Lu-PSMA-617 was administered with a fixed activity of 7.4 GBq per cycle at 6-week intervals for four cycles. Two additional cycles could be administered based on patient response, tolerance, and residual disease with the same fixed activity without interim dosimetry. In the phase 3 trial, Pluvicto increased median overall survival by 4 months, median progression-free survival by 5.3 months, and reduced prostate-specific antigen by more than 50%, Sartor et al. (2021).

As a result of the FDA approval of ^177^Lu-DOTATATE and ^177^Lu-PSMA-617, the availability of RPT agents targeting cancer and radiation treatment centers offering RPT has increased significantly. As of June 2023, the number of healthcare systems offering Lutathera and Pluvicto in the United States has increased to 285 and 102, respectively, AAA (2023).

### 3.2 Contemporary developments

With the approval of these newly developed radiopharmaceuticals, numerous studies and investigations are being conducted on attaching and delivering ^177^Lu to different types of tumors using a variety of tagging mechanisms and physiological processes. Table 1 provides a comprehensive list of clinical studies in the United States with different treatment conditions under investigation with ^177^Lu as a metal-ligand.

**TABLE 1:** Radiopharmaceuticals based on ^177^Lu used in active, completed, and recruiting studies in the USA, Trials (2023).

Interventions	Number of studies	Treatment conditions
177Lu-DOTATATE (Lutathera)	25	GEP NETs
177Lu-PSMA-617 (Pluvicto)	7	PSMA-positive mCRPC
177Lu-J591	6	PSMA-positive mCRPC
177Lu-DOTATOC	2	NETs
177Lu-PSMA-I&T	2	PSMA-positive mCRPC
177Lu-DOTA-HH1 (Betalutin)	2	B-cell non-Hodgkin lymphomas
177Lu-PNT2002	2	mCRPC
177Lu-DOTA-ABM-5G	1	advanced/metastatic PDA
177Lu-DOTA-EB-TATE	1	metastatic NETs
177Lu-CC49	1	PSMA-positive mCRPC
CTT1403	1	PSMA-positive mCRPC
177Lu-DOTA-JR11	1	NETs
177Lu-NeoB	1	GRPR-positive tumors
177Lu-girentuximab	1	metastatic ccRCC
GD2-SADA:177Lu-DOTA	1	GD2-positive Solid tumors
177Lu-FAP-2286	1	FAP-positive Solid tumors
177Lu-Ludotadipep	1	mCRPC
177Lu-rhPSMA-10.1	1	PSMA-positive mCRPC

Many tumors contain cancer-associated fibroblasts (CAFs), which express high levels of fibroblast activation protein (FAP). As a vital component of the tumor microenvironment, FAP plays a crucial role. The goal of targeting FAP is to disrupt the tumor microenvironment and inhibit cancer cell growth. Several approaches have been developed to target FAP, including small molecule inhibitors, monoclonal antibodies, and radiopharmaceuticals, Zboralski et al. (2020).

The radiopharmaceutical candidate FAP-2286 has a targeting peptide that binds to FAP and a location where radioactive isotopes can be attached (Lu-177 for therapeutic purposes and Ga-68 for imaging). Several studies have shown promising results for ^177^Lu-FAP-2286, Zboralski et al. (2022); Baum et al. (2021), and Clovis Oncology is conducting a clinical trial using FAP-2286 on solid tumors, Trials (2023).

FAP inhibitor (FAPi) molecules have also generated interest in imaging, but their potential therapeutic applications are still under investigation. The biodistribution, pharmacokinetics, and dosimetry of the FAPi agents [^177^Lu]Lu-DOTA.SA.FAPi and [^177^Lu]Lu-DOTAGA.(SA.FAPi)_2_ were compared in a trial with ten patients. The findings supported [^177^Lu]Lu-DOTAGA.(SA.FAPi)_2_, which internalized more quickly, had a higher affinity, retained in the tumor for a longer period, and cleared non-target organs faster, Ballal et al. (2021). FAPi-46 is another radiotracer that received significant research attention in the last 5 years, and just recently, ^177^Lu has been used as a suitable radiometal for FAPi-46, Liu et al. (2021).

Aside from the clinical trial evaluations mentioned above, other prominent targeted receptors utilize ligands conjugated with ^177^Lu. The targeting of human epidermal growth factor receptor 2 (HER2) in breast cancers and their metastasis (bone, lung, and lymph nodes), Bhusari et al. (2016), Carbonic Anhydrase IX (CAIX), a protein overexpressed in hypoxic tumor cells, Muselaers et al. (2016), and cholecystokinin2 receptor (CCK2R) in medullary thyroid cancer, Rottenburger et al. (2019), are active areas of research, and listed in Table 2.

**TABLE 2:** Other prominent ^177^Lu-based radiopharmaceuticals under investigation not included in the clinical trials search and their targets.

Radiopharmaceutical	Target	Treatment conditions
177Lu-DOTA.SA.FAPi	FAP	Solid tumors
177Lu-DOTAGA.(SA.FAPi)2	FAP	Solid tumors
177Lu-FAPi-46	FAP	Solid tumors
177Lu-trastuzumab	HER2	Breast Cancer
177Lu-brentuximab	CAIX	Hypoxic tumors
177Lu-PP-F11N	CCK2R	Medullary Thyroid Cancer

Multimerization produces molecules with high diagnostic and therapeutic potential. Multimeric ligands are formed by tethering several monomeric molecules with similar or different functions. A high degree of selectivity and binding affinity can be achieved by adjusting parameters such as linker length and flexibility, scaffold and backbone insertions, and recognition of ligands and receptors, Carlucci et al. (2012). Some radiopharmaceuticals like ^177^Lu-EB-PSMA-617, ^177^Lu- DOTA-EB-TATE, and CTT1403 are designed to achieve a higher tumor uptake with a dual targeting molecule. Besides the primary targets (PSMA or SSTR), they also target albumin serum to increase the blood circulation time of the radiopharmaceutical and therefore achieve a higher tumor uptake. The downside to the albumin binding is an increase in hematologic toxicity and an increase in radiation dose to healthy organs, Ling et al. (2019); Zang et al. (2020); Thakur et al. (2021).

### 3.3 Dose limiting considerations

^177^Lu is excreted from the body through the kidneys, which are highly vascular organs that filter and eliminate the substance. In addition, ^177^Lu-DOTATATE binds to SSTR expressed on renal cells, leading to radiation damage and nephrotoxicity. Garske et al. (2012), estimates the mean kidney absorbed dose for patients receiving Lutathera treatment to 2.2 Gy/GBq. The study also found that patients who received higher doses of Lutathera had an increased risk of renal toxicity, including proteinuria and decreased glomerular filtration rate (GFR). In most cases, the kidneys excrete ^177^Lu; however, tubular reabsorption can accumulate ^177^Lu in the kidney cortex. One of the most significant factors limiting the effectiveness of ^177^Lu-octreotate is renal toxicity caused by renal accumulation. In light of this, it is necessary to understand the biodistribution and dosimetry of ^177^Lu in individual patients, Larsson et al. (2012).

Bone marrow produces blood cells, including white blood cells, red blood cells, and platelets. An evaluation of Lutathera treatment showed that patients absorbed a mean dose of 0.86 Gy/GBq to the bone marrow. Radiation-induced damage to the bone marrow can lead to myelosuppression, a condition characterized by decreased blood cell counts and an increased risk of infection and bleeding associated with high bone marrow doses, Sabet et al. (2013). Lutathera’s dose-limiting OARs are kidneys and bone marrow because of their high radiosensitivity and SSTR expression.

The TD_5/5_ limit of 23 Gy for whole-kidney irradiation (the dose that would cause a 5% chance of a complication within 5 years of treatment) was adapted from previous clinical experience with external beam treatments. In addition, it was based on preclinical and clinical data from other radiopharmaceuticals targeting SSTR receptors, Staanum et al. (2021). Dawson et al. (2010), did an extensive study on radiation-related injuries and identified dose-volume constraints with an estimated risk of less than 5% for 30% volume of bilateral kidneys receiving more than 23 Gy.

As PSMA is expressed on the surface of salivary acinar cells, radiation-induced xerostomia or dry mouth may be a potential concern in PSMA-targeted therapy. In external beam radiation, the mean dose to the parotid is limited to less than 25 Gy to ensure a less than 20% chance of losing long-term salivary function. Cell membrane damage and impaired signaling are associated with acute dry mouth, while progenitor cell death limits the regeneration process in chronic dry mouth effects. A dry mouth can adversely induce dental caries, swallowing difficulties, and speech changes. However, these symptoms gradually subside over six to 24 months of dose administration, Sartor et al. (2021). Another potential concern is radiation-induced nephrotoxicity or kidney damage. PSMA is expressed in proximal renal tubule cells and vascular cells. Although the kidney dose in Lu-177-based PSMA therapy is similar to that in PRRT with Lu-DOTATATE, a protocol for nephroprotection has not yet been developed. It has also not been shown that amino acid co-infusion reduces kidney radiation exposure, Jackson et al. (2022); Mix et al. (2021). Other rarely occurring severe side effects of Lu-177 PSMA therapy include increased bleeding, nausea, and fatigue.

## 4 Current status of patient-specific dosimetry

Dosimetry is limited by variations in methodologies for establishing administered activity in routine clinical practice. Few prospective studies show superior outcomes for dosimetry-based treatments over empirical dosing.

**FIGURE 3:**
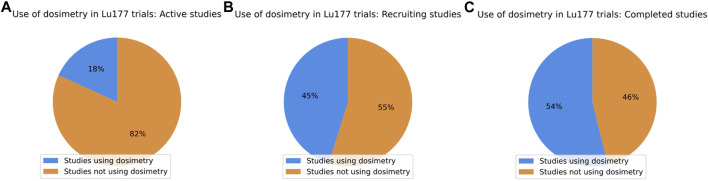
Distribution of dosimetry usage on ^177^Lu trials in the United States. **(A)** Only two of 11 active studies had dosimetry implemented. **(B)** Only 14 out of 31 recruiting studies reviewed used dosimetry. **(C)** 13 completed studies were identified, 7 of which implemented dosimetry.

A filtered search using ^177^Lu, Dosimetry, and Absorbed Dose provided information for active, recruiting, and completed studies from the clinical trials website. This yielded 57 studies directly related to ^177^Lu radiopharmaceuticals in the United States. The studies were analyzed according to interventions and treatment conditions and are displayed in Table 1. The data for this table was obtained by searching public websites. It was downloaded in a TXT, or CSV file format, evaluated for the required information with Python, and uploaded to Mendeley Data, Aguirre and George (2023). Out of the 31 recruiting studies, dosimetry is only a primary or secondary outcome measurement in 14. There are 11 active studies, with only two conducting dosimetry measurements. Furthermore, seven of the 13 completed studies used dosimetry as a significant outcome measure, Trials (2023). It can be seen from Figure 3 that dosimetry has been of limited interest in different clinical trials under investigation. These trials aim to improve approved treatments, expand ^177^Lu-based treatment options, and introduce combinations of RLT and chemotherapy or immunotherapy.

### 4.1 Development of patient-specific dosimetry

Radiopharmaceutical dosimetry and external beam radiotherapy dosimetry are similar in that they involve the calculation of the radiation dose delivered to the patient. Still, they differ concerning the source and distribution of radiation, dosimetry imaging methods, and calculations performed to determine radiation dose, Ljungberg and Gleisner (2016); Wahl et al. (2021).

#### 4.1.1 Source of radiation

Radiopharmaceutical therapy delivers radiation through interventional procedures or infusions of therapeutic agents targeted at tumors. In external beam radiotherapy, a radiation source is a machine that directs radiation beams at the patient from outside the body.

#### 4.1.2 Distribution of radiation

In radiopharmaceutical dosimetry, the radiation is distributed throughout the body and is targeted to specific areas by metabolic activity, as shown by imaging techniques. In external beam radiotherapy, the radiation is focused on a particular body site, typically a tumor, through specialized equipment such as a linear accelerator.

#### 4.1.3 Dosimetry imaging

Imaging methods used for radiopharmaceuticals include planar scintigraphy, single photon emission computed tomography (SPECT), or positron emission tomography (PET). In addition to computed tomography, magnetic resonance, and portal imaging are used in external beam radiotherapy.

#### 4.1.4 Dosimetry calculations

In radiopharmaceutical dosimetry, the radiation dose is calculated based on the activity of the radioactive material in the body, the patient’s body weight, and organ-specific imaging data. In external beam radiotherapy, the radiation dose is calculated based on the intensity and duration of the radiation beams, the patient’s body shape, and the tumor’s location.

### 4.2 Optimization of treatment through dosimetry

Dosimetry is an essential aspect of ^177^Lu treatments, allowing for personalized medicine, improved safety and efficacy, monitoring of the therapy, and compliance with regulations, Wahl et al. (2021).

#### 4.2.1 Personalized treatment

Dosimetry allows accurate measurements of the radiation dose administered to the patient, which could be used to tailor to the individual patient’s treatment needs. This can help optimize therapeutic benefits while minimizing the risk of adverse effects.

#### 4.2.2 Improved safety and efficacy

Dosimetry allows an accurate calculation of the radiation dose to the target area and surrounding normal tissue, which can help minimize the risk of adverse effects. This is particularly important for radiopharmaceuticals containing ^177^Lu, which have a relatively short half-life and emit beta and gamma radiation. Dosimetry allows precise targeting of radiation dose to specific areas of the body being treated, which can help maximize the therapeutic benefit of ^177^Lu.

#### 4.2.3 Monitoring the therapy

Dosimetry allows measuring the radiation dose over time, which can be used to monitor therapy during the course of treatment and adjust treatment as necessary.

#### 4.2.4 Compliance with regulations

Dosimetry is a requirement of certain regulatory bodies for using ^177^Lu and other radiopharmaceuticals, to ensure that the therapy is being used safely and effectively, Konijnenberg et al. (2021); Lassmann et al. (2021).

### 4.3 Standard techniques used for patient-specific dosimetry

During the last 10 years, as a result of the availability of integrated functional-anatomic imaging systems such as PET/CT and SPECT/CT that compensate for factors that degrade images, the ability to perform patient-specific dosimetry and quantitative imaging of therapeutic radionuclides has been dramatically improved, St James et al. (2021). Radionuclide treatments based on ^177^Lu use several dosimetry methods. Dosimetry evaluations can be performed using a one- or two-time point protocol based on the number of scans performed post-drug administration, making them more suitable for clinical implementation, Peters et al. (2023); Hanscheid et al. (2017). Calculating time and tissue-specific dose factors is also possible using a single post-treatment SPECT/CT scan, Jackson et al. (2020), Jackson et al. (2022). EANM published guidelines for the dosimetry of PSMA labeled with ^177^Lu and ligands targeting somatostatin receptors, Kratochwil et al. (2019). Table 3 gives an overview of the advantages and disadvantages of different methods.

**TABLE 3:** Advantages and disadvantages of different dosimetry techniques.

Technique	Pros	Cons
Planar scintigraphy	Easily accessible and cost effective	Lack of anatomical information results in relatively low accuracy
	Suitable for routine clinical use	Organs with a high ^177^Lu uptake, such as the kidneys, may receive an underestimated dose
	Easy to perform and simple to understand	Inaccurate when organs overlap
SPECT	Better spatial resolution than planar scintigraphy	Specialized equipment is required
	Determine the activity distribution in three dimensions accurately	Complex and time-consuming process compared to planar scintigraphy
	An improved dose estimation method	Patient motion can affect measurements
		Inaccurate when organs overlap due to Partial Volume Effect (PVE)
Time activity curve analysis	Improves the accuracy of time activity curves for each organ	More frequent blood sampling and imaging is required
	Estimation of radiation absorbed dose is better than planar scintigraphy and SPECT	Results may vary between different institutions according to calculation methods
Monte Carlo simulation	Simulating radiation behavior in tissues with high accuracy	Computationally demanding and time-consuming
	Calculates the dosimetry accurately	Expertise and specialized software are required
		Simulation sensitivity to input parameters
Hybrid imaging	Enhances accuracy by combining anatomical and functional information	Equipment and expertise are required
	Better radiation absorbed dose estimation than planar scintigraphy or SPECT alone	Time-consuming and costly imaging
		Inaccurate when organs overlap

#### 4.3.1 Planar imaging

Following administration of the ^177^Lu radiopharmaceutical, planar imaging is performed on the patient. Based on these images, the Medical Internal Radiation Dose (MIRD) formalism can be used to estimate the absorbed dose in the tumor and normal tissues, Siegel et al. (1999).

#### 4.3.2 Single photon emission computed tomography imaging

SPECT imaging provides 3D information on the distribution of radiopharmaceuticals within a patient. SPECT images can estimate the absorbed dose using the MIRD formalism or voxel-based dosimetry using the activity uptake in the tumor and normal tissues, Stabin et al. (2005); Ljungberg and Gleisner (2016).

#### 4.3.3 Positron emission tomography imaging

With PET imaging, smaller lesions can be visualized using a better spatial resolution than SPECT imaging. Additionally, using voxel-based dosimetry, PET images can estimate the absorbed dose, Sgouros et al. (2004).

#### 4.3.4 Hybrid imaging

SPECT/CT and PET/CT are examples of hybrid imaging combining the functional and anatomical characteristics of two or more imaging modalities. In this way, dosimetry calculations can be improved by better localization of tumors and normal tissues, Beyer et al. (2000); Ljungberg and Gleisner (2016).

#### 4.3.5 Time activity curves

Serial blood sampling and imaging determine radioactivity in the blood pool and the target organs over time. When these data are analyzed, a curve can be created showing the activity concentration in the target organ over time. This curve can be fitted with a mathematical model to calculate the effective half-life and the radiopharmaceutical residence time within the organ. Based on this information, the absorbed dose is estimated, Hindorf et al. (2010); Lawhn-Heath et al. (2022); Ljungberg and Gleisner (2016).

Ultimately, dosimetry is concerned with determining the dose an organ or tumor has absorbed because biological effects can be predicted more accurately by the dose absorbed rather than the activity administered. To calculate the absorbed dose, there are three basic approaches: dose factor (S value)-based calculation, dose-point kernel convolution, and Monte Carlo (MC) radiation transport simulation, Danieli et al. (2022).

#### 4.3.6 Dose factor (S value)-based calculation

For dose factor (S value)-based estimates at the organ level, the MIRD schema is widely used, Capala et al. (2021); St James et al. (2021).

#### 4.3.7 Dose-point kernel convolution

This method uses a dose point kernel that represents the radial absorbed dose in a homogeneous water medium when an isotropic point source is located at the center. TERMA (Total Energy Released per Unit Mass) and the Kernel will compute the dose, Lee et al. (2019); St James et al. (2021).

#### 4.3.8 Monte Carlo simulation

Computer programs are used to simulate radiation transport through a medium using Monte Carlo simulations. Considering a patient’s specific anatomy and heterogeneity, MC can provide a detailed assessment of absorbed dose in tumor and normal tissues, Sandstrom et al. (2020); St James et al. (2021).

### 4.4 Commercially available software

Several commercial software programs are available for ^177^Lu dosimetry, each using different scientific principles. Development of scientific tenets, standardization, and commercially available software are briefly discussed here. This information is provided solely for academic purposes. It is not intended to be commercially biased, even if we discuss some specific vendor details.

The Society of Nuclear Medicine established a committee in 1965, MIRD, to standardize dosimetry calculations with improved radionuclide emission and radio pharmacokinetic data. MIRD Pamphlet No. 1, first published in 1968, attempts to provide a unified approach to internal dosimetry. It has been updated several times since then. Currently, the MIRD Primer from 1991 is the most widely known version. MIRD Pamphlet No. 21, published in 2009, provided a comprehensive nomenclature to bridge the formalism differences between the MIRD Committee and the International Commission on Radiological Protection (ICRP), MIRD (2023); Bolch et al. (2009).

RADAR™ (RAdiation Dose Assessment Resource) has been used for internal dose estimation since 2003 in OLINDA/EXM™ 1.0. The RADAR method calculates voxel-based absorbed doses by multiplying the time-integrated activity by the dose factors for organs with significant radiopharmaceutical uptake. The absorbed dose in a target organ is estimated as the product of the number of nuclear transitions in the region multiplied by the dose factor. This depends on the mass of the target region, the energy of radiation, the absorbed fraction, and the number of radiation emissions per nuclear transition. This process is repeated for all identified sources and targets to obtain a total dose for all target organs. RADAR developed OLINDA/EXM™ version 2.0 software which uses voxel-based realistic human computational phantoms for dose estimates. Like MIRD and ICRP, RADAR calculates internal doses but uses different terms and symbols, Stabin (2018); Stabin and Siegel (2018).

In addition to ^90^Y-microsphere SIRT, PLANET Dose (DOSIsoft™) is FDA-approved and CE-marked for other isotopes, including ^177^Lu for 3D RPT dosimetry. Multiple image sets and VOIs can be rigidly or deformably registered using DICOM-compatible images from diverse imaging modalities. Various methods can be used to integrate time-activity curves, and S values for voxels are used to calculate 3D doses. This software has been validated using MC simulations and OLINDA/EXM™, Capala et al. (2021).

The GE™ Healthcare Dosimetry Toolkit is an application that calculates organ time-integrated activities (TIA) and mean absorbed doses from organ volumes and time-activity curves. The system supports serial whole-body planar scanning, SPECT/CT scanning, or hybrid imaging. SPECT workflows include image reconstruction, serial scan registration, segmenting organs, calculating volumes, activities, and TIA, and segmenting target organs. OLINDA/EXM compatible or Microsoft Excel files are provided as output, Capala et al. (2021).

MIM SurePlan MRT™ calculates the absorbed dose based on voxel-based calculations. A dose map and DVH curve are presented for analysis in MIM SurePlan MRT using the voxel s-value (VSV) schema in MIRD Pamphlet No. 17. With MIM Software; SPECT images can be quantitatively reconstructed with CT-based attenuation correction, scatter correction, and resolution recovery, Capala et al. (2021). In addition, an FDA-approved artificial intelligence platform is used to segment organs and tumors and calculate absorbed doses. Various radionuclides can be measured with the software, and their quantitative accuracy has been tested, MIM (2023). SPECT images are rigidly registered and then merged to create composite images. It is possible to do SPECT/CT dosimetry using multiple scans over an extended period, a hybrid approach, or a single scan. Several models calculate TIA, including trapezoidal integration with exponential terms for extrapolation, Capala et al. (2021).

Torch™ is Voximetry™’s software package incorporating a manual or automated dosimetry workflow. It can register images, propagate contours, model kinetics, and calculate radiation transport using parallel processing. A proprietary MC algorithm is included in the software that is accelerated by the graphics processor. To use Torch, the user must import DICOM images and ROIs for at least one imaging time point. Torch’s deformable registration algorithms can propagate contours across other time points for multiple-time-point dosimetry, or users can import their ROIs, Capala et al. (2021).

Voxel dosimetry™ is a CE-marked software and FDA 510(k) cleared developed by Hermes Medical Solutions™, which provides patient-specific dosimetry for clinically used radiopharmaceuticals. It uses MC simulations to obtain accurate dose results and can also perform single-time-point dosimetry. Hermes Medical Solutions also markets Organ Dosimetry™. From a nuclear medicine image, it analyzes activity concentrations inside organs and tumors over time. Through seamless integration with OLINDA/EXM^®^, it generates absorbed dose tables for all volumes of interest, Hermes (2023).

## 5 The importance of patient-specific dosimetry

The variability in patient response to treatment is one of the critical challenges in radiopharmaceutical therapy. The use of patient-specific dosimetry plays a crucial role in addressing this problem. It involves measuring and quantifying the radiation dose absorbed by target tissues and normal organs. This information can be used to optimize treatment planning and dose administration to maximize efficacy and minimize toxicity. After treatment with ^177^Lu-tagged radiopharmaceuticals, the patient is expected to have an improved quality of life and longevity. There is a possibility that patients can experience a recurrence of cancer or develop metastasis in other parts of their body after receiving radiation treatments. Due diligence must be applied to ensure no radiation treatment overlaps at treatment sites or nearby critical organs unless the previous dosage is accounted for. A dose summary from a composite treatment plan is generated when a patient returns for re-treatment with external beam radiation therapy to achieve this purpose. If personalized dosimetry is lacking in RPT, it will limit our ability to achieve the highest level of precision when treating cancer with various methods of radiation therapy.

Multiple dosimetry studies show a significant variation in ^177^Lu uptake in patients’ kidneys and tumors, Satterlee et al. (2015); Ballal et al. (2021); Hanscheid et al. (2017). Individualization of treatments by modifying the number of cycles and administered activity remains a topic of tremendous interest in radiation oncology and the nuclear medicine community. Post-injection dosimetry can be performed to ensure safety and evaluate absorbed doses, Arveschoug et al. (2020). For safety and to assess treatment efficacy, dose verification after each cycle of Lutathera treatment is desirable. Recent studies indicate that a single SPECT CT study performed after 96 h of administration will provide comprehensive data for dosimetric analysis, Hanscheid et al. (2017). There is still much to explore regarding clinical evaluations and, more importantly, quantification of absorbed dose after treatment (to targets and critical organs) for better personalized ^177^Lu treatments.

The VISION and NETTER-1 trials’ prescribing dose per cycle of 7.4 GBq is intended to limit the cumulative kidney dose to <23 Gy. Significant differences in kidney doses between individuals administered the fixed doses demonstrate the importance of patient-specific dosimetry and treatment planning, Larsson et al. (2012); Jackson et al. (2022). It has become increasingly common for new studies and trials to adhere blindly to the dose limits for critical organs, followed by EBRT experience. Radiation doses delivered to the target tissue and normal organs at risk can differ significantly depending on factors such as kidney function, tumor burden, location, and vascularity. Absorbed dose escalation studies are essential to determine the exact limit for renal radiation-absorbed doses. By adhering to the EBRT limit, some patients receiving RPT may receive an inadequate tumor dose, affecting their efficacy, Wahl et al. (2021). With advances in radiation oncology, optimizing treatment outcomes and improving patient care through patient-specific dosimetry will become increasingly necessary, Danieli et al. (2022); Lawhn-Heath et al. (2022). Ensuring a safe and effective treatment that utilizes accurate and individualized dosimetry is essential. Tailored radiation doses can be administered safely and effectively, improving treatment outcomes and reducing toxicities, Alsadi et al. (2022); Sjogreen Gleisner et al. (2022). Continued research is necessary to enhance the efficacy of radiopharmaceutical therapy and refine dosimetry techniques. Dr. Stephen Graves states, “It is important to remember that radiation-absorbed dose, not radiopharmaceuticals, is the drug,” MIM (2023).

### 5.1 Advantages of patient-specific dosimetry

Personal dosimetry is especially important for radiopharmaceuticals tagged with ^177^Lu, which has a relatively short half-life of 6.7 days, meaning that the radiation dose will decrease rapidly over time. Dosimetry allows for accurate measurement of the radiation dose at the time of treatment, allowing adjustments to be made in subsequent treatments if necessary. ^177^Lu treats conditions that include neuroendocrine tumors and metastatic prostate cancer, while many other trials and developmental work that require precise radiation doses targeting specific body areas are pursued. Dosimetry allows for accurate calculation of the radiation dose in these target areas, ensuring that therapeutic benefit is maximized while minimizing the risk of adverse effects. ^177^Lu is becoming more commonly used as a therapeutic agent, so dosimetry is essential to ensure its safety and effectiveness. ^177^Lu decays by emitting both beta and gamma radiation. Beta particles have low penetration power and release most of their energy at a short distance. Dosimetry accurately measures the radiation dose delivered to normal tissue and targets, which can minimize adverse effects. Dosimetry is crucial to ensure effective and safe usage of radiopharmaceuticals containing ^177^Lu by allowing accurate measurement and calculation of radiation dose delivered to the patient, precise targeting of the radiation to specific areas of the body, and minimizing the risk of adverse effects, Brans et al. (2007).

### 5.2 Combination of EBRT and RPT requires improved dosimetry

EBRT and RPT are two methods of radiation therapy used to treat cancer. Ionizing radiation causes irreparable DNA strand breaks, the primary mechanism in both treatments. Biologically, these two modes produce profoundly different effects; the two modes are administered differently and have dissimilar dosage distributions, dose rates, and cytotoxicity. EBRT involves toxicity to the tissues closest to the tumor, whereas ^177^Lu radionuclide therapy is characterized by toxicity to normal tissues based on its pharmacokinetics, Abbott et al. (2021). Researchers found that ^177^Lu-DOTATATE and EBRT induce similar radiobiological mechanisms but to a different extent and with variable kinetics, including DNA damage responses, Delbart et al. (2022).

The combination of EBRT and RPT may offer significant benefits in treating cancer. In combination with systemic therapy, EBRT can effectively debulk dominant tumor masses and control subacute cancers. Studies show that EBRT and ^90^Y SIRT can be safely administered to HCC patients, Wang et al. (2018). The combined dose from both sources could be calculated using the biologically effective Dose (BED), a quantitative measure of the biological effect of radiotherapy treatment. EBRT and RPT have non-overlapping toxicity profiles, but interactions may lead to super-additivity, where one agent sensitizes the tumor to the other. In addition, one modality may enhance another’s toxicity by causing molecular changes in cancer cells. The combination of EBRT and RPT has been challenging because no dosimetric framework exists to link the spatiotemporal pattern and energy deposited by each to their combined biological effects, Abbott et al. (2021). However, precedence exists in gynecological cancers, where brachytherapy and external beam treatments are jointly used for disease management, keeping track of cumulative biological doses.

Radiotherapy aims to destroy as many tumor cells as possible to eliminate the tumor without inflicting excessive damage on the patient. Unfortunately, tumor cells are not all alike. Hence, selecting the correct agent, technique, and appropriate treatment dose determines the efficiency of radiotherapy. The clinical benefit of RPT depends on delivering an accurate radiation dose to cancer cells early enough in their life cycle to achieve tumor control. ^177^Lu-tagged radioligands have the potential to improve cancer diagnosis and treatment significantly, and further studies in this area are warranted.

### 5.3 Future-proofing with advanced dosimetry

^177^Lu-based therapy could be enhanced by personalized dosimetry, allowing customized treatment regimens based on individual patient characteristics. Dosimetry research has improved the understanding of ^177^Lu biodistribution and has helped improve cancer therapies based on ^177^Lu. Different organs and tissues receive different radiation doses based on the biodistribution and clearance of ^177^Lu, as shown by dosimetric studies, Alsadi et al. (2022); Sjogreen Gleisner et al. (2022). During ^177^Lu therapy, there can be significant variations in radiation dose received by different organs and tissues. The amount of ^177^Lu absorbed by tumors is impacted by their location, and individual characteristics add to this variability.

Dosimetry calculations require accurate analysis and imaging because errors in measurement of ^177^Lu distribution can result in inaccurate radiation dose deposited. Mathematical modeling and computational advances in hybrid imaging, such as SPECT/CT and PET/CT, have substantially improved dosimetry calculations. Developing advanced computational models, including Monte Carlo simulations, has remarkably advanced dosimetry modeling in recent years. As a result of these models, the radiation dose received by various organs and tissues can be predicted more accurately, optimizing treatment regimens for individual patients. A systematic approach based on patient-specific dosimetry is necessary for tailored radiation treatment for each patient. Using single-time point imaging study is scientifically proven to yield reasonably accurate dosimetry output and is convenient for patients and clinicians, Brosch-Lenz et al. (2023).

## 6 Conclusion

During phase I of clinical trials for any novel RPT, dosimetry should be an integral component. Dosimetry helps determine organ-absorbed dose while respecting the maximum tolerated amount and recommends a phase II dose. Safety requires establishing exposure doses for normal organs and the entire body. In phase II studies, dosimetry can determine a treatment’s dose-response relationship and efficacy, Pandit-Taskar et al. (2021).

According to EANM procedure guidelines for ^177^Lu-labeled PSMA-ligands, it has been established that red marrow has a tolerance limit of 2 Gy (single exposure), the kidneys have a tolerance limit of 28–40 Gy, and the salivary glands have a tolerance limit of 35 Gy based on publications that corrected for the masses of individual organs. It is recommended that exposures to target volumes be individually planned and verified and doses to non-target volumes be kept as low as reasonably achievable. The European Directive 2014/59/EU (translated into national regulations on 6 February 2018) requires individualized planning and verification of doses to target volumes, Kratochwil et al. (2019); Lassmann et al. (2021). The conservative approach of using a generic and low tolerance of 23 Gy kidney dose and other organ dose limits used in the VISION and NETTER-1 trials should be revisited. A large part of the determination of this threshold dose in the trials came from studies using ^177^Lu-DOTATATE and data from external beam radiation therapy without proper scrutiny.

Radiopharmaceutical therapy must use patient-specific dosimetry to establish a meaningful relationship between adverse events and absorbed doses to vulnerable organs and accurately quantify the dose delivered to the intended targets. This approach will provide a proper framework for targeted radionuclide therapies widely used in early-stage cancer patients. Incorporating dosimetry into RPT clinical trials may lead to customized treatment cycles with optimal individualized dosage schemes. Combination treatments beyond what is currently accepted could be designed and implemented safely in clinical practices.
