# Corrigendum: Developments in ^177^Lu-based radiopharmaceutical therapy and dosimetry

**DOI:** 10.3389/fchem.2024.1410803

**Published:** 2024-04-17

**Authors:** Siju C. George, E. James Jebaseelan Samuel

**Affiliations:** ^1^ Radiation Oncology Department, Miami Cancer Institute, Baptist Health, Miami, FL, United States; ^2^ Department of Physics, School of Advanced Sciences, Vellore Institute of Technology, Vellore, India

**Keywords:** ^177^Lu, absorbed dose, patient-specific dosimetry, dose calculations, imaging, calculation methods, clinical trials, contemporary developments

In the published article, the wrong author was included in the **Correspondence**. The correct corresponding author is E. James Jebaseelan Samuel (ejames@vit.ac.in).

In the published article, the department and school were erroneously omitted from affiliation 2. The correct affiliation appears above.

The authors apologize for this error and state that this does not change the scientific conclusions of the article. The original article has been updated.

